# Propofol and sufentanil may affect the patients’ sleep quality independently of the surgical stress response: a prospective nonrandomized controlled trial in 1033 patients’ undergone diagnostic upper gastrointestinal endoscopy

**DOI:** 10.1186/s12871-017-0341-3

**Published:** 2017-03-31

**Authors:** Ming Lei, Peng Zhang, Yunfei Liu, Fangfang Fu, Ling Ye, Tao Zhu

**Affiliations:** 1 Department of Anaesthesiology, West China Hospital, Sichuan University, Chengdu, Sichuan Province People’s Republic of China; 2 Current address: Department of Anaesthesiology, AVIC 363 Hospital, Chengdu, Sichuan Province People’s Republic of China; 3 Department of Anaesthesiology, Sichuan Academy of Medical Sciences and Sichuan Provincial People’s Hospital, Chengdu, Sichuan Province People’s Republic of China; 4 Department of Pain Management, West China Hospital, Sichuan University, Chengdu, Sichuan Province 610041 People’s Republic of China

**Keywords:** Propofol, Anaesthetics, Sleep, Pittsburgh Sleep Quality Index

## Abstract

**Background:**

It is unknown whether sedative per se contributes to the postoperative sleep disturbance. Diagnostic upper gastrointestinal endoscopy (UGE) is a minimally invasive procedure which is not likely to cause tissue trauma and pain. The purpose of this study was to evaluate the sleep quality of patients undergoing routine (without sedative) diagnostic UGE or UGE with sedative, before, 1 week, and 1 month after the procedure.

**Methods:**

One thousand and thirty-three patients undergoing UGE were enrolled. Patients chose sedative or without sedative. Propofol and sufentanil were administered to the sedative group, not allowed for the routine group. The Pittsburgh Sleep Quality Index (PSQI) was measured before, 1 week and 1 month after the procedure.

**Results:**

Five hundred and ten patients were enrolled in the sedative group and 523 in the routine group. One week after the procedure, patients in the sedative group showed significantly higher PSQI scores (worse sleep quality) than the baseline PSQI scores (*p* < 0.001), but there was no significant change for the routine group in the same period (*p* = 0.096). One month after the procedure, there was no significant difference in PSQI scores between the two groups compared with the baseline values (*p* = 0.358 for sedative group, *p* = 0.161 for routine group). There were also no significant difference in the PSQI scores between the two groups in the entire 1 month follow-up period (*p* = 0.885).

**Conclusions:**

The sedative group showed impaired sleep quality 1 week after diagnostic UGE. Propofol and sufentanil may independently affect the sleep quality of patients after sedative of diagnostic UGE for only one week.

**Trial registration:**

This study is registered on Chinese Clinical Trial Registry (IDChiCTR-OCH-13003128). Registered 2 April 2013.

**Electronic supplementary material:**

The online version of this article (doi:10.1186/s12871-017-0341-3) contains supplementary material, which is available to authorized users.

## Background

Although the function of sleep is unknown, it is no doubt that a normal sleep pattern and cycle is important to maintain the normal function of physiological and mental processes [1]. Previous studies have shown that inpatients, particularly postoperative patients, for example cardiac surgery [2], hip and knee arthroplasty [3], major abdominal surgery [4], even laparoscopic cholecystectomy [5] often complained about sleep disturbance. Surgical stress response, sedative and postoperative pain are the main factors that may influence the postoperative sleep [6]. However, previous studies always reported those three factors together, so it was not known whether the sedative would independently affect postoperative sleep disturbance.

Diagnostic upper gastrointestinal endoscopy (UGE) is a minimal invasion procedure which can be performed with or without sedative. Propofol, opioids or midazolam are commonly used to provide the sedation to facilitate patients’ tolerance [7]. Also, it is well-known that diagnostic UGE is not likely to cause tissue trauma and patients seldom complain of pain after this procedure. By the advantage of these factors, this study attempts to estimate whether sedative independently contribute to the postoperative sleep disturbance without the confounding effects of surgical stress response and postoperative pain.

With higher degree of internal homogeneity, overall consistency and clinical validity than any other test available, the PSQI (Pittsburgh Sleep Quality Index), originally described by Buysse in 1989, has established itself as the main tool for the assessment of quality of sleep [8]. Generally speaking, the reporting period of PSQI is 1 month, but the PSQI questionnaire was used for short-term evaluation for 1 week in our study. Broderick et al once have evaluated the accuracy of PSQI across different lengths of reporting periods (3-, 7- and 28-days). They found there is no significant difference of PSQI scores among 3-, 7- and 28- days reporting period [9]. Besides, many previously studies investigated the short-term sleep quality using PSQI [2, 10–16]. Therefore, PSQI can be also administered with confidence for weeklong reporting periods for between-subject analyses. In this study, we rely on PSQI questionnaire to evaluate the sleep quality of diagnostic upper gastrointestinal endoscopy participants before, 1 week and 1 month after the procedure.

## Methods

### Patients

**Fig. 1:**
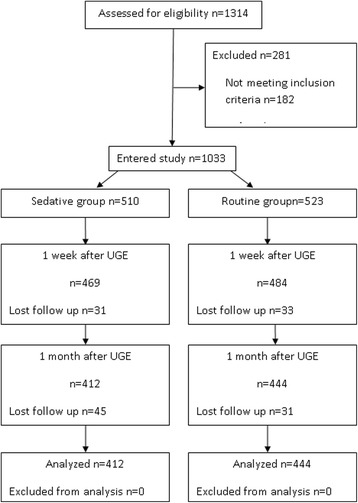
Study flowchart. UGE = diagnostic upper gastrointestinal endoscopy

The study was approved by the institutional ethics committee of West China Hospital Sichuan University (Chengdu, China) and registered on Chinese Clinical Trial Registry (ID ChiCTR-OCH-13003128). Written informed consent about the study protocol was obtained from each patient preoperatively. Patients who decided diagnostic UGE under sedative were allocated to the sedative group, while other patients allocated to the routine group (without sedative). One thousand and thirty-three patients, ≥18 years, American Society of Anaesthesiologists (ASA) physical status classification I–II, undergoing outpatient diagnostic UGE were enrolled to the study (Fig. 1). Exclusion criteria included known sleep disorders, use of sedatives or hypnotics, alcohol abuse (more than 35 units per week), expected compliance problems (known psychiatric disease, difficulty in reading or speaking in Chinese) or undergoing sedative (general or local) or surgery during the 1 month follow-up period.

### Diagnostic upper gastrointestinal endoscopy procedure

Standard monitoring including electrocardiograph (ECG), pulse oximeter (SPO_2_), and noninvasive blood pressure (BP) were applied. Two minutes before endoscopy, patients were pre-oxygenated (Fraction of inspiration O2, FiO_2_ = 1.0) by face mask and oxygen was delivered (FiO_2_ = 0.33) for the following procedure. For the sedative group, anaesthetic management was standardized. After pre-oxygenation, sedative was administered with sufentanil 3-5 μg over 5 to 10 s and propofol (1% Diprivan™; AstraZeneca Japan, Osaka, Japan) 1.0 mg · kg^–1^intravenously. Supplemental doses of 0.5 mg/kg propofol were administered when the patient was conscious or body movement was found [17]. In contrast, no anaesthetics were administered to the patients in the routine group and the patients maintained conscious throughout the procedure. The diagnostic UGE was performed with a video endoscope by the experience gastroenterologist. Atropine (0.005-0.01 mg/kg) or ephedrine (0.1-0.2 mg/kg) would be respectively administered IV, when HR was less than 60 bpm or systolic blood pressure was less than 80 mmHg or 80% of its baseline. Patients who achieve Modified Post anaesthesia Discharge Scoring System (PADSS) score of 9 or more were considered to be ready for discharge [18].

### Quality of sleep

The sleep quality was assessed using the 19-item PSQI (Pittsburgh Sleep Quality Index) questionnaire (Additional files 1 and 2) [8, 9]). The PSQI have 7 components including sleep latency, sleep duration, habitual sleep efficiency, sleep disturbances, daytime dysfunction, and use of sleeping medications which range 0–3, respectively. The global PSQI score ranging from 0 to 21 is generated by summing up all the seven component scores, where 0 indicates no difficulty and 21 is severe difficulty in all areas. A global cut-off score of PSQI greater than 5 is used to distinguish poor sleepers from good sleepers.

All patients were required to evaluate their sleep quality as the baseline when they make an appointment on the Endoscopy Center of West China Hospital 3-5 days before the diagnostic UGE. At the time of the follow-up visit (1 week and 1 month after procedure), all patients were asked to assess their sleep quality via telephone by an investigator who was blinded to the assigned groups.

### Sample and statistical analysis

Based on our pilot study data from our 150 patients, the mean PSQI scores were 5.24 for the sedative group and 4.82 for the routine group with a standard deviation (SD) of 2.11. A sample size of 796 (398 in each group) allowed the detection of a 20% difference between the two group, with an α of 0.05 (two tailed) and a β of 0.20, power of 0.8. To account for 20% attrition, a sample size of 956 (478 in each group) was selected.

Data was analyzed with SPSS Version 13.0 (SPSS Inc., Chicago, IL). Mann-Whitney *U* test or *χ* ^2^ test was used to compare the two groups with respect to the background characteristics such as age, sex, ASA and baseline PSQI scores. Linear mixed model was used to evaluate the PSQI score differences between the two groups and changes after post-endoscopy time. Bonferroni correction was made when necessary to correct for multiple test. *P* < 0.05 was considered statistically significant.

## Results

Five hundred and ten patients were enrolled for sedative group and 523 for routine group. All patients participated in the PSQI questionnaire before endoscopy. Thirty-one (6.1%) patients in the sedative group and 33(6.3%) patients in the routine group were lost for the 1 week follow-up after endoscopy. Forty-five (9.6%) patients in the sedative group and 31 (6.4%) in the routine group were lost for the 1 month follow up. Twenty-two (4.3%) patients in the sedative group and 15 (2.9%) patients in the routine group were excluded because another sedative was administered during the 1 month follow-up period (Fig. 1). Finally, 856 (82.9%) patients were analyzed. Demographic variables were similar among the two groups (Table 1). There was no significant difference between the two groups for the pathological findings (*p* = 0.172, Table 2).

**Table 1:** Patient characteristics in two groups

	Sedative group (N = 412)	Routine group (N = 444)	*p* value
Age(year)	60(18-80)	61(19-81)	0.801
Sex(male/female)	211/201	229/215	0.915
ASA(I/II/III)	156/178/78	192/190/62	0.096

Values are given as frequencies or median (range)

**Table 2:** Endoscopic and pathological findings of the diagnostic upper gastrointestinal endoscopy between two groups

	Sedative group, n (%)	Routine group, n (%)
Negative findings	123 (29.85%)	139 (31.31%)
Gastritis	173 (41.99%)	193 (43.47%)
Peptic ulcer	72 (17.48%)	58 (13.06%)
Malignancy	20 (4.85%)	34 (7.66%)
Others	24 (5.83%)	20 (4.50%)
Total	412 (100%)	444 (100%)

**Fig. 2:**
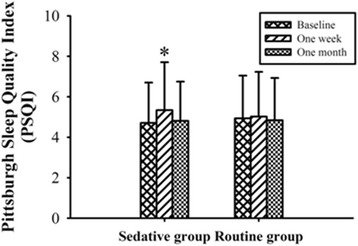
PSQI (Pittsburgh Sleep Quality Index) scores at 3 time points in the two groups (*p < 0.05 versus Baseline PSQI). There were no significant differences with regard to the baseline PSQI scores (*p* = 0.097) between groups. There was no significant differences in the routine group (*p* = 0.096) in the same period. The PSQI scores returned to the baseline values in both groups, 4.81 ± 1.94 in the sedative group (*p* = 0.358) and 4.84 ± 2.09 in the routine group (*p* = 0.161) 1 month after the diagnostic UGE

PSQI scores were 4.71 ± 1.99 in the sedative group and 4.93 ± 2.11 in the routine group before diagnostic UGE. There were no significant differences with regard to the baseline PSQI scores (*p* = 0.097) between groups. Sedative group had a global PSQI score of 5.34 ± 2.37 and 5.02 ± 2.21 in the routine group, 1 week after diagnostic UGE. The sedative group patients showed higher PSQI scores 1 week after diagnostic UGE than the baseline values (*p* < 0.001), but there was no significant differences in the routine group (*p* = 0.096) in the same period. The PSQI scores returned to the baseline in both groups 1 month after the diagnostic UGE (4.81 ± 1.94 in the sedative group, *p* = 0.358, and 4.84 ± 2.09 in the routine group, *p* = 0.161). However, there was no significant difference in PSQI scores between two groups in the entire 1 month follow-up period (*p* = 0.885). The PSQI scores in the two groups at the individual questionnaire time points were shown in the Fig. 2.

## Discussion

The current study demonstrated that only patients undergoing sedative diagnostic UGE showed significant impaired sleep quality compared with baseline values 1 week after procedure. To our knowledge, this is the first study attempted to investigate the independent effect of sedative on the sleep quality of patients undergoing diagnostic UGE. In this study, propofol and sufentanil may independently affect the sleep quality for only 1 week after diagnostic UGE.

Several studies have shown the similar results about impaired postoperative sleep quality. For example, in a non-randomized controlled trial study, Yilmaz et al investigated the self-report sleep quality and objective sleep parameters in 85 patients undergoing coronary artery bypass grafting (CABG) surgery and 40 healthy humans [2]. The authors observed significantly impaired sleep quality and sleep architecture in CABG patients 1 week after surgery but matched healthy humans did not show this problem during the same follow-up period. Our study also showed significantly diminished sleep quality 1 week after sedative diagnostic UGE. Probably because cardiac surgery was a more invasive surgery procedure, the CABG patients showed more serious of impaired sleep quality than our patients. However, the quality of sleep returned to the preoperative baseline values 1 month after CABG, so did the diagnostic UGE.

The injury of surgery followed by a complex stress response has been proposed as an important cause of postoperative sleep disturbances. The stress response involving endocrine-metabolic system and an inflammatory response may last for a few days or even weeks [19]. Moreover, the magnitude of the surgical procedure was associated with the severity of sleep disturbances [6, 20]. For example, probably as a result of more extensive surgical trauma, the patients following the open cholecystectomy suffered worse sleep quality than patients following the laparoscopic cholecystectomy [21]. Postoperative pain is another reason for sleep disturbances after surgery. Since diagnostic UGE was not likely to cause tissue injury and pain after the procedure, the potential effect of surgical stress response and postoperative pain as the co-contributors to postoperative sleep disturbances would not make any confusion in this study. We successful evaluated the independent effect of sedative or anaesthetics on postoperative sleep disturbance in the diagnostic UGE patients.

Psychological factors including anxiety and negative mood states have been known to contribute to the sleep disturbances [22]. Patients’ psychological condition can be easily affected by the state of illness, especially malignant disease. Since no significant difference was found with regard to endoscopic and pathological findings of the diagnostic UGE in the sedative group compared with the routine group, we believed that the effect of psychological factors on the sleep quality were comparable between the sedative and routine group.

Although inducing sleep-like EEG slow waves [23], propofol recently have been demonstrated to significantly change the sleep architecture with increase inN3 sleep and total abolishment of rapid-eye-movement (REM) in health humans [24, 25]. Opioids use, whether acute (when used to provide sedation or sedative) or chronic, have been shown to disturb the sleep pattern [26]. For example, some studies have shown that morphine significantly altered sleep architecture with increase of N2 sleep and reduction of slow wave sleep (SWS) in health humans [27, 28]. REM and SWS are generally associated with the restorative processes in the body (especially the energy restoration) [29]. Therefore, we presume that the reduction of REM and SWS might be the most possible reasons that sedative patients showed subjective impaired sleep quality 1 week after diagnostic UGE.

A limitation of our design was the absence of randomization. Therefore evident as well as unknown confounders might have influenced the outcomes. Upper gastrointestinal endoscopy is an invasive procedure and some patients can’t tolerance this procedure [30]. We considered it unethical to randomize the participants regardless of their desires. In fact this study was a real world study.

## Conclusion

In conclusion, 1033 patients (510 for sedative group VS. 523 for routine group) were enrolled to evaluate the sleep quality of patients undergoing routine (without sedative) diagnostic UGE or UGE with sedative, before, 1 week, and 1 month after the procedure. The sedative group showed impaired sleep quality only for 1 week after diagnostic UGE. Therefore, propofol and sufentanil may independently affect the sleep quality of patients undergoing diagnostic UGE.

## Additional files

Additional file 1: PSQI questionnaires for 1 week. (PDF 111 kb)

Additional file 2: PSQI questionnaires for 1 month. (PDF 92 kb)

## Abbreviations

ASA: American Society of Anaesthesiologists
BP: Noninvasive blood pressure
CABG: Coronary artery bypass grafting
ECG: Electrocardiograph
FiO2: Fraction of inspiration O2
PADSS: Post anaesthesia discharge scoring system
PSQI: Pittsburgh Sleep Quality Index
REM: Rapid-eye-movement
SD: Standard deviation
SPO2: Pulse oximeter
SWS: Slow wave sleep
UGE: Upper gastrointestinal endoscopy
