# Supplementary material for: Propofol and sufentanil may affect the patients’ sleep quality independently of the surgical stress response: a prospective nonrandomized controlled trial in 1033 patients’ undergone diagnostic upper gastrointestinal endoscopy
Source: BMC Anesthesiol. 2017 Mar 31;17:53. doi: 10.1186/s12871-017-0341-3 (PMC5374607; doi:10.1186/s12871-017-0341-3)
Supplement: Supplementary file 2 — PSQI questionnaires for 1 month. (PDF 92 kb) [file 12871_2017_341_MOESM2_ESM.pdf]

Pittsburgh Sleep Quality Index (PSQI)

Instructions: ***The following questions relate to your usual sleep habits during the past MONTH only. Your answers should indicate the most accurate reply for the majority of days and nights in the past MONTH.***

During the past month,

1. When have you usually gone to bed? ___________________
2. How long (in minutes) has it taken you to fall asleep each night? ___________________
3. When have you usually gotten up in the morning? ___________________
4. How many hours of actual sleep did you get that night? (This may be different than the number of hours you spend in bed) ___________________

5. During the past month, how often have you had trouble sleeping because you…	Not during the past month (0)	Less than once a week (1)	Once or twice a week (2)	Three or more times a week (3)
a. Cannot get to sleep within 30 minutes				
b. Wake up in the middle of the night or early morning				
c. Have to get up to use the bathroom				
d. Cannot breathe comfortably				
e. Cough or snore loudly				
f. Feel too cold				
g. Feel too hot				
h. Have bad dreams				
i. Have pain				
j. Other reason(s), please describe, including how often you have had trouble sleeping because of this reason(s):				
6. During the past month, how often have you taken medicine (prescribed or "over the counter") to help you sleep?				
7. During the past month, how often have you had trouble staying awake while driving, eating meals, or engaging in social activity?				
8. During the past month, how much of a problem has it been for you to keep up enthusiasm to get things done?				
	Very good (0)	Fairly good (1)	Fairly bad (2)	Very bad (3)
9. During the past month, how would you rate your sleep quality overall?				

**Component 1**	**#9 Score**	**C1_______**
**Component 2**	**#2 Score (≤ 15min (0), 16-30 min (1), 31-60 min (2), >60 min (3))**<br>**+ #5a Score (if sum is equal 0=0; 1-2=1; 3-4=2; 5-6=3)**	**C2_______**
**Component 3**	**#4 Score (>7(0), 6-7(1), 5-6(2), <5 (3)**	**C3_______**
**Component 4**	**(total # of hours asleep)/(total # of hours in bed) x 100**<br>**>85%=0, 75%-84%=1, 65%-74%=2, <65%=3**	**C4_______**
**Component 5**	**# sum of scores 5b to 5j (0=0; 1-9=1; 10-18=2; 19-27=3)**	**C5_______**
**Component 6**	**#6 Score**	**C6_______**
**Component 7**	**#7 score + #8 score (0=0; 1-2=1; 3-4=2; 5-6=3)**	**C7_______**

Add the seven component scores together_________Global PSQI Score _________
